# Dr. Simon

**Published:** 1912-02

**Authors:** 


DR. SIMON, a bacteriologist of Zurich, Switzerland, died January 5, 1912, from septicaemia due to the bite of an infected mouse. He was endeavoring to prepare a serum. "Peace hath her heroes, no less than war." Or will the antis regard this as a righteous retribution?

